# Perceived Burden and Quality of Life in Caregivers of Patients with Schizophrenia in Saudi Arabia’s Eastern Province: A Cross-sectional Study

**DOI:** 10.2174/0117450179314013240417105321

**Published:** 2024-08-22

**Authors:** Feras Al-Awad

**Affiliations:** 1 Department of Psychiatry, College of Medicine, Imam Abdulrahman Bin Faisal University, Dammam, Saudi Arabia

**Keywords:** Burden, Caregivers, Family, Quality of life, Saudi Arabia, Schizophrenia

## Abstract

**Background and Aims:**

Family Caregivers (FCGs) of patients with schizophrenia (PwS) may face unanticipated sources of stress and responsibility, which can negatively impact their quality of life (QoL). This study aimed to assess FCGs' QoL and the impact of clinical characteristics of patients and sociodemographic factors on their QoL.

**Patients and Methods:**

A cross-sectional questionnaire-based study surveyed 340 FCGs from outpatient clinics of PwS in two large psychiatric hospitals in Saudi Arabia's eastern province using a convenience sampling approach. We used the Adult Carer Quality of Life (AC-QoL) scale, which has eight subscales and 40 items, to assess QoL. AC-QoL is translated into Arabic in this study.

**Results:**

The study included 216 FCGs, with 127 (58.8%) being men, 117 (54.2%) being over 45 years old, 91 (42.1%) being a sibling of a PwS, and 82 (38%) being a parent of a PwS. The mean score in our sample was 78.2 ± 21.24 out of 120, indicating mid-range QoL. Lower QoL was associated with more time spent in caregiving per day, a lower educational level of FCG, and recent admission of PwS to an inpatient unit.

**Conclusion:**

PwS FCGs have a mid-range QoL. FCGs reported a moderate financial burden and low levels of support from healthcare professionals. FCG's QoL and stress can be reduced through healthcare providers, participation in a community support group, and addressing an FCG's in an individual setting.

## 1 INTRODUCTION

Schizophrenia is a chronic mental illness characterized by the presence of multiple symptoms, including delusions, hallucinations, negative symptoms, and concurrent emotional and behavioral disturbances [1]. Schizophrenia, like other severe mental disorders, such as bipolar disorder and schizoaffective disorder, causes significant result disruptions in many areas of life, including interpersonal relationships, social functioning, employment, education, and self-care [2].

Family caregivers (FCGs) play an important role in the care of patients with schizophrenia (PwS) in many societies [3]. FCGs provide emotional support, behavioral management, assistance with daily living, financial assistance, and interaction with healthcare professionals [4]. FCGs may encounter unexpected sources of stress, such as the patient's abnormal behaviors and thoughts. PwS's quality of life (QoL) has improved due to deinstitutionalization and reliance on community services and FCGs. FCGs may bear a significant burden, mainly when there are insufficient psychological resources or health services [5, 6]. Long-term caregiving can deplete family energy and cause despair, helplessness, sadness, and the onset or exacerbation of mental disorders in other family members [7]. Over the last decade, research has shifted away from patients and toward FGC. Caregiving responsibilities can have a negative impact on a carer's physical health [8], financial burden [9], employment rates [10], and QoL.

QoL refers to physical and emotional well-being, psychological and social well-being, achievement of personal goals, financial stability, and the ability to perform daily activities without difficulty [11]. Previous research has shown that FCGs of people with psychiatric disorders, particularly those with PwS, have lower QoL scores than carers of people with other psychiatric illnesses [10]. The reduced QoL experienced by FCGs of PwS can be attributed to emotional responses to the disease, difficulties in managing the disturbed behavior of family members, disruption of daily household routines, limitations in social and recreational activities, and financial constraints [12].

Over the last decade, researchers have focused on the burden and QoL of caregivers of PwS. There has been little research on caregiver burden and QoL among Arab caregivers of people with disabilities.

In Saudi Arabia, research in this field is limited. The published studies focused on patients with mental disorders or severe mental illness rather than caregivers of PwS [13]. Furthermore, there has been very little quantitative and qualitative research in Arab countries, with only a few studies conducted, primarily in Egypt and Jordan [14-16]. According to the studies, the caregiver burden ranged from moderate to severe, and it was influenced by coping style, social support, and psychological well-being.

Given the potential impact of differences in healthcare systems, community resources, social systems, and other cultural elements on family burden [17-20], it is critical to assess the applicability of findings across diverse cultural contexts [21]. The primary goal of this cross-sectional study, which was conducted in outpatient clinics of two psychiatric facilities in Saudi Arabia's eastern province, was to assess the extent of caregiver burden and QoL among FCGs of PwS. The secondary goal of the study is to look into the potential impact of sociodemographic factors and patient characteristics on QoL and caregiver burden.

## 2 MATERIAL AND METHODS

### 2.1 Sample and Setting

A questionnaire was used in this quantitative, cross-sectional study of PwS FCGs. All procedures were performed in accordance with ethical standards and regulations. G*Power 3.1.9.6 [22] was used to conduct a *post hoc* power analysis to determine the minimum sample size required to test the study hypotheses. The sample size needed to achieve 80% power for detecting a medium effect at a significance level of *p* = 0.05 in our study was *N* = 180 (*f* = 0.25). We approached 340 family caregivers and received 216 responses (63% response rate). As a result, the sample size of *N* = 216 obtained is adequate for testing the study hypotheses.

Participants were randomly drawn from psychiatry outpatient clinics at King Fahad Hospital of University in Khobar, Saudi Arabia, and Eradah Mental Health Complex, a community mental health hospital in Dammam.

A retrospective review of medical records was carried out at two large psychiatric hospitals in eastern Saudi Arabia (King Fahad University Hospital in Khobar and the Eradah Complex in Dammam). The study was carried out between June and October 2023. Each year, the facility receives approximately 105,000 outpatient clinic visits. Patients with schizophrenia were identified using the institution's electronic medical record system. We obtained outpatient visit data from the medical records department for the 12 months prior to the start of our investigation (June 2022 to June 2023). We looked for a diagnosis of schizophrenia using ICD-10 coding (F22). In total, 987 patients were identified. The medical records were reviewed in two stages. During the first stage of chart review, the investigators searched for schizophrenic patients who were actively visiting clinics. Out of 987 patients identified, the study sample was randomly selected using research randomizer software (randomizer .org) with a target number of 375, assuming a 50% response rate. We then completed the second stage of chart review and called the caregiver to screen for those who met the inclusion criteria. A primary caregiver was defined as the person who met most of the patient's daily needs—(physical, emotional, financial, social, nurturing, and others). Inclusion criteria included (a) being a primary FCG of a family member who met schizophrenia criteria based on the Diagnostic and Statistical Manual of Mental Disorders, 5th Edition (DSM-5) criteria, (b) caregiving more than >7 h per week or more than >1 h per day, (c) family member aged 18 yr, (d) care-recipient of any age, and (e) literacy in Arabic and the ability to complete a survey questionnaire. Participants were included in the study if they met the inclusion criteria outlined above, and the investigators informed them of the study's objectives and details, the voluntary nature of their participation, and the strict confidentiality of their responses. The survey was purposefully designed to be self-administered. However, before leaving participants to complete the questionnaire, the researcher provided an overview and answered any questions they may have had. Participants responded to the questions *via* a secure online survey platform (QuestionPro). The survey was carried out in Arabic, and the data were gathered anonymously.

### 2.2 Survey Instruments

The questionnaire was divided into three sections. In the first section of the survey, the FCG's socio demographic information was collected, which included gender, age group, education level, employment status, household income, relationship to PwS, and time spent in caregiving. The second section of the survey asked about the patient's sociodemographic and clinical characteristics, such as age, gender, illness duration, number of lifetime admissions, admissions in the previous 6 months, and whether the patient was taking long-acting injectable antipsychotics or oral medications.

The Adult Carer Quality of Life (AC-QoL) scale was used to assess the QoL of adult carers in the survey's third and final segment [23]. Joseph (2012) developed this 40-item scale, which includes eight subscales that assess the following: support for caring, caring choice, caring stress, financial matters, personal growth, sense of value, ability to care, and carer satisfaction. Each AC-QoL subscale and the total score with a higher number indicates a higher QoL. The highest possible score is 120. The total AC-QoL score is divided into three categories: “low” (0–40), “medium” (41–49), and “high” (80+).

We used the methods demonstrated by Sousa *et al*. [24] to translate the original English AC-QoL scale to Arabic. First, two bilingual and bicultural translators translated the AC-QoL into Arabic. Both translators were well-versed in medical terminology, instrument construction, and cultural and linguistic differences. The questionnaire's items, as well as the instructions and responses, were all translated. A committee combined two translated versions into one Arabic version (synthesis I). The Arabic questionnaire was back-translated into English by two bilingual, bicultural translators. After resolving ambiguities and contradictions, a committee examined all questionnaire versions and agreed on the prefinal version (synthesis II). The preliminary version was tested on 20 nonstudy participants. The Arabic AC-QoL scale was finalized after reviewing the results and resolving any outstanding issues. The Arabic scale was highly reliable (0.93, overall scale), whereas the original English scale had a Cronbach's *α* coefficient of 0.94.

### 2.3 Statistical Analysis

SPSS version 29 (IBM Corp., Armonk, New York) was used for all data analyses. Statistics such as descriptive statistics and inferential statistics were used. Continuous variables were summarized using means and standard deviation (*SD*). Categorical variables were described using frequency and percentages. The AC-QoL scores were treated as a continuous dependent variable, whereas the sociodemographic information was treated as an independent variable. We analyzed the distribution of the study variables using the kolmogrov-smirnov test. The means of AC-QoL were compared using independent sample t-tests for two independent variables. For more than three independent variables, a one-way analysis of variance (ANOVA) was used to investigate variations in mean AC-QoL scores in relation to other demographic variables. We used ANOVA to compare sample means and determine statistical significance because each group follows an approximately normal distribution. When we run ANOVA, we add the AC-QoL total score to the “dependent” list. All other variables were added to the “factor” list as independent variables. For instance, factors such as patient relationship and caregiving level were considered independent. The computation was conducted using SPSS. The results of the ANOVA test included the sum of squares, degree of freedom, mean square, F, and level of significance. The F statistic compares the variability between and within groups. The F-statistics was calculated using the following formula: (F = Variance between groups/Variance within groups). A P-value of less than 0.05 was considered statistically significant in all tests. To assess significance, SPSS compares the calculated F-statistic to the critical F-value from the F-distribution at a specific significance level (*e.g*., α = 0.05). If the p-value is less than the specified significance level, reject the null hypothesis and conclude that there are significant differences between the group means. The hypothesis was to determine whether caregiver burden is associated with QoL among FCGs of PwS. The null hypothesis implies that there is no association between the two variables, whereas the alternative hypothesis suggests that there is an association.

The Imam Abdulrahman Bin Faisal University's Institutional Review Board (IRB-2023-01-118) approved all procedures in this study as ethical. All participants electronically signed a consent form before answering any questions.

## 3 RESULTS

Our study included a total of 216 FCGs. We noticed that caregivers of PwS were more likely to be men and from lower socioeconomic backgrounds. FCGs in our study were 58.8% men, 54.2% over 45, 83.3% with a high school diploma, and 45.8% employed. Although the majority of the PwS in our study were young adults (aged 18-40), we found that siblings are more likely than parents to take on caregiving responsibilities. The PwS's siblings accounted for 42% of FCG, with the remainder being a parent (38%), spouse (9.7%), or child (10.2%). According to caregivers' reports, 59.3% of PwS were men; people aged 18–40 yr made up 50.5% of the sample; 73.1% of the patients had a duration of illness of more than 8 yr; 48.1% of the patients had one to three admissions in their lifetime, whereas those who had never been admitted made up only 26.4% of the sample. Long-acting injectable antipsychotics were used by one-third of the patients (36.6%). Table **1** shows the sociodemographic characteristics of FCGs, and Table **2** shows the sociodemographic and clinical characteristics of PwSs.

Table **3** shows the mean and SD of each subscale on the AC-QoL scale and the overall score. Given that our sample's mean score was 78.2 ± 21.24 out of 120, the QoL total score indicates mid-range QoL. A higher score indicates better QoL and less caregiving burden. Each subscale is assigned a maximum of 15 points, with 0 being the lowest possible score. The scores of the eight QoL subscales varied significantly, with “support for caring'” (mean 7.32 ± 0.31) and “money matters” (mean 8.39 ± 3.99) being the most affected. These findings indicate that families perceive a lack of emotional support and information about how to deal with the illness from healthcare professionals and that their needs are not fully met. The questions about “money matters” reflect their concern about the future of their financial life and/or the availability of sufficient funds to support the person with the illness. FCGs reported lower personal growth from caregiving (mean 8.92 ± 3.82). The questions in this domain investigated whether caregivers believed caring increased their stress tolerance, taught them more about themselves, and provided them with many positive experiences. Interestingly, FCGs had low levels of caring stress (mean 12.03 ± 3.68) and high levels of “care satisfaction” (mean 11.31 ± 3.25).

In the relationship between FCG's sociodemographic variables and QoL score, total time spent in caregiving had a statistically significant impact on QoL score, (*F*= 5.30, *p* < 0.01). FCGs who worked 6–12 h per day had a lower mean (69.25 ± 19.96) than those who worked 1–6 h per day (81.15 ± 19.38, *p* < 0.01).

Using ANOVA, we attempted to investigate whether the age, gender, and type of relationship of the caregivers would predict the perceived level of QoL. Opposite to what we expected, we did not find that all these variables to have any significant impact on QoL. Whether the caregiver was a sibling, spouse, or parent, the difference in mean scores was statistically insignificant. We expected females would report a lower level of QoL since caregiver burden is generally more reported by women than men, but the difference in mean scores was statistically insignificant (F=0.432, p=0.512).

Furthermore, results from the ANOVA test showed that the level of education of FCGs has a statistically significant impact on the total QoL score but not the socioeconomic status. FCGs with a high school diploma or higher had a higher QoL score than those without (mean difference = 8.59, *SE* = 3.95, *p* < 0.05). Therefore, the household monthly income and caregiver employment status did not impact the level of QoL. In terms of the relationship between QoL score and clinical and sociodemographic variables of PwS, PwS admitted in the previous 6 months had a higher mean QoL score (*t* = 3.74, *p* < 0.001) than those who had not been admitted in the last 6 months. PwS variables, such as illness duration, number of lifetime admissions, and PwS sex, had no statistically significant impact on QoL scores. Table **4** contains additional information. Table **4** contains additional information.

## 4 DISCUSSION

The primary findings of the present study indicate that FCGs of PwS have QoL in the moderate range. They scored the lowest on the AC-QoL scale in terms of finances and support from healthcare professionals. In contrast, FCGs performed well in QoL measures assessing stress and caregiving satisfaction. The amount of time spent providing care, the caregiver's level of education, the clinical instability of PwS, and recent admissions to an inpatient unit all had a negative impact on QoL.

As far as we know, our study is the first study in Saudi Arabia to use and validate an Arabic version of the AC-QoL scale, which measures QoL and carer burden for those who care for people with disabilities. According to the literature, family members who care for people with schizophrenia are consistently overburdened by their responsibilities [25-27]. A recent systematic review found that chronic mental illnesses, particularly schizophrenia, are associated with a moderate-to-severe carer burden [26]. Our participants reported a moderate financial burden, which is consistent with the findings of other studies [12, 28, 29]. Direct costs, such as medications and appointments, or indirect costs, such as house rent, mortgages, grocery shopping, and transportation, typically contribute to the financial burden. The financial burden is most likely due to indirect costs because we recruited participants from governmental hospitals where treatment is free. Because schizophrenia typically begins in childhood, many of those patients will lose the ability to care for themselves or even work. In our experience, those government and healthcare professionals usually have low-paying jobs due to a lack of a college education or low productivity. The lack of government assistance contri-butes to these families' severe financial hardship. A systematic review from different European countries estimated that the average proportion of indirect costs of total costs was 44% [30]. A study on 404 patients in five European countries revealed that the most common worries of relatives were about the patient’s health and their own future and financial position [31].

We found that caregivers were dissatisfied with the emotional and practical support provided by healthcare professionals. This finding highlights the importance of psychological and educational interventions in providing FCGs with the resources they require to support a person with schizophrenia. FCG of PwS requires formal and informal support from family, friends, the government, and healthcare professionals to ensure their well-being. A multidisciplinary approach is necessary to provide the best care possible to PwS and their caregivers (social worker, psychologist, occupational therapist, and psychiatrist). The quality of caregivers can be improved with the assistance of local community services, such as support groups and the Schizophrenia Society. To enhance the quality of care for these patients with severe mental illnesses, such as schizophrenia, the mental healthcare system should prioritize the needs of FCGs. They should also have access to services, such as respite care, which gives caregivers a temporary break from their responsibilities. Schizophrenia is a crippling mental illness that places a significant emotional and financial burden on the patient's family. A strong support system for FCGs will most likely result in faster rehabilitation from mental illness and addiction, a lower risk of death, less reliance on health care services, a lower rate of rehospitalization and relapse, higher medication adherence, better interpersonal functioning, and stronger families [32].

According to our findings, caring stress was low, and care satisfaction was relatively high. Caregivers of recently admitted PwS had higher levels of care stress (mean difference = −2.16, *t* = −3.43, *p* = 0.001) and lower levels of care satisfaction (mean difference = −2.05, *t* = −3.095, *p* = 0.003) than caregivers of PwS who had not been admitted to an inpatient unit. The fact that most people in our sample (81.5%) had not been hospitalized in the previous 6 months and that all caregivers were recruited from outpatient clinics could explain why the sample had low levels of caring stress and high levels of care satisfaction. According to studies [25, 33] when a patient has an acute relapse and needs to be hospitalized, the caregiving burden increases. An extensive cross-sectional study conducted in the United Kingdom, Germany, and Spain revealed that caregivers who reported hostile and violent patient behaviors experienced a significantly greater burden [34]. Previous qualitative and quantitative systematic reviews have shown that FCGs of PwS with more severe psychotic symptoms are at a significantly higher risk of experiencing a heavy burden [4, 35, 36]. Additional research indicates that problematic or disruptive behavior in patients, as well as positive and negative symptoms of schizophrenia, are associated with high levels of burden [37-39]. A study conducted in Italy found that families characterized by high emotional expression (EE) are more likely to report subjective burden. [40]. Thus, it is advisable for healthcare professionals to investigate the existence of elevated EE.

In terms of the relationship between caregivers' sociodemographic variables and QoL score, the caregiver's educational degree had a statistically significant impact on the QoL score. Higher education confers some socioeconomic advantage and facilitates exposure to broader social networks and resources, which may mitigate the negative effects of caregiving. Previous research, including a systematic review, found a negative relationship between socioeconomic status and the burden of caregiving [26, 41-43].

The total amount of caregiving time had a statistically significant effect on QoL. Caregivers who worked longer hours were more negatively impacted. Our findings are consistent with previous research that found a link between patient contact time and burden [44, 45]. For example, a study on 680 Dutch FCGs of PwS, which used an Involvement Evaluation Questionnaire, found that relatives who had less than 1 h per week of contact with the patient showed less distress compared with relatives with more than 1 h per week (mean score 12.4 *vs*. 13.6, *t* = 3.36, *p* < 0.001). We should remember that the immediate family size in Saudi culture is larger than in the Western world. Some authors have suggested that a larger family size reduces the impact of increased caregiver-patient contact time on the care burden [46]. As a result, extra assistance is more readily available to those large families. The average Saudi family has 6.4 members [47], ranging from 5.5 to 8.4.

We found no statistically significant relationship between carers' monthly household income, gender, age group, or employment status and their QoL score. Our findings support previous research that found no relationship between the caregiver age and burden level [27, 41, 43]. We believe that aging alone cannot affect perceived QoL or burden because other factors, such as resilience, family commitments, retirement, health status, and the size of social support, may be associated with aging.

Furthermore, there were no significant gender differences in QoL total score between female and male caregivers. This finding is consistent with other studies [25, 41, 43], which found no significant relationship between gender and caregiving burden. On the contrary, Yükü and Derleme [26] reported that the caregiver burden was higher for females than for males. Male caregivers outnumbered female caregivers in our sample by a significant margin (58.8% to 40.3%). Although the literature suggests that caregivers are more likely to be women than men [48], we found no evidence of this in our study. This finding could be explained by the fact that in Saudi Arabian culture, the role of caregiving for PwS is assigned based on the patient's gender, with male patients receiving care from male caregivers and female patients receiving care from female caregivers. Another cultural explanation is that people with disabilities frequently require assistance with vocational activities and managing life issues outside the home, such as rent, finances, work, and social connections [49]. In Saudi Arabia, male caregivers are expected to meet the needs of PwS by providing financial support, taking the lead in scheduling appointments and transportation and handling mortgage payments, whereas female carers are expected to focus on housework [50].

Neither the duration of the patient's illness nor the number of lifetime admissions had a statistically significant effect on the caregivers' overall QoL in our study. We found that disease duration is not significantly related to caregiver burden, which is consistent with previous research [51]. We hypothesize that the longer the illness, the longer the caregiver, the more tolerant, resilient, and stress-resilient the caregiver becomes. Although caregiving is frequently associated with significant distress and burden, there have been accounts of its positive effects [36, 52]. Caring for people with disabilities has been linked to various positive outcomes for caregivers [53], including increased satisfaction, pride, competence, and self-worth [54, 55]. Furthermore, caring for people with special needs can improve caregivers' stress-coping skills [56]. Positive health outcomes have also been linked to active coping [38]. Nonetheless, a systematic review study that included 39 articles found more extensive empirical evidence suggesting the negative effects of prolonged psychosis on family members of people with severe mental illness [57].

Our study has some limitations. The use of a convenience sampling approach method may have introduced some selection bias. Additionally, most PwS in our sample were from outpatient settings; carers of patients receiving inpatient treatment are more likely to have lower QoL, but they are underrepresented in this study. Another limitation is that we only enrolled participants from two hospitals, limiting our findings to the hospital-based population. The adaptability and applicability of the AC-QoL in the present study to measure caregiver burden is a major limitation. Furthermore, AC-QoL has only been tested on caregivers of patients with severe mental illness in a few studies. The Arabic-translated scale's psychometric properties have not yet been tested. While the study emphasizes the role of caregiver sociodemographic factors and patients' clinical characteristics on QoL, it does not look into causal relationships, such as the level of anxiety depression among caregivers or the type of symptoms in PwS. Furthermore, due to the nature of our study being a cross-sectional observational study, it is unable to establish a causal relationship between variables. Additionally, the presence of confounding factors may affect the results, limiting its generalizability to other populations or settings. Moreover, the lack of randomization prevents the allocation of risk factors for the outcome of interest by chance. Finally, the sample size (*N* = 216) is relatively small.

## CONCLUSION

Despite its limitations, this is the first study to look at caregiver burden and QoL among caregivers of PwS, to the best of our knowledge. At best, our participants' QoL was moderate. Carers reported a moderate financial burden and low support from healthcare professionals. The number of hours spent providing care, the caregiver's education level, and recent hospitalization all had a negative impact on QoL. Physicians should inquire about the caregivers' support system and promote the delegation of responsibilities among family members, especially for those who dedicate more than 12 hours to caregiving. In addition, individuals who have recently been discharged from an inpatient unit should be given priority to receive additional psychosocial support, as they are more susceptible to experiencing a lower QoL. Community support groups and psychoeducation can help caregivers improve their QoL and reduce stress. It is critical to assist carers in learning to cope with the stresses of caregiving. We propose increasing emotional and social support to lighten the load on carers. Our findings also emphasize the importance of focusing on the specific challenges faced by carers with lower levels of education and longer tenure in the field. Considering that caregivers with lower academic education exhibited lower QoL, it is recommended that they be prioritized in psychoeducational interventions. Furthermore, given that a significant percentage of caregivers in our study expressed concern regarding their financial prospects, it is imperative to implement governmental initiatives aimed at providing assistance to family caregivers. Future research should include measuring patients' psychopathology and functioning to understand better how it affects patients' QoL and the stress placed on carers. Furthermore, levels of social support, depression and anxiety, carers' coping mechanisms, and self-sufficiency must be assessed and analyzed in conjunction with caregiver burden and quality of care.

## Figures and Tables

**Table 1:** Sociodemographic characteristics of caregivers.

Character	N (%)	
**Gender**	-	
Male	127 (58.8)	
Female	87 (40.3)	
**Age group (years)**	-	
< 45	98 (45.4)	
> 45	117 (54.2)	
**Relationship with the patient**	-	
Parent	82 (38)	
Sibling	91 (42.1)	
Spouse	21 (9.7)	
Child	22 (10.2)	
**Education level**	-	
< High school	35 (16.2)	
≥ High school	180 (83.3)	
**Employment status**	-	
Employed	99 (45.8)	
Unemployed	117 (54.2)	
**Monthly income of household**	-	
< 5000 SR	53 (24.5)	
5000—10,000 SR	69 (31.9)	
10,000—20,000 SR	43 (19.9)	
> 20,000 SR	31 (14.4)	
**Time spent in caregiving (per day)**	-	
1—6 hours	116 (53.7)	
6—12 hours	44 (20.4)	
> 12 hours	56 (25.9)	

**Table 2:** Sociodemographic characteristics of patients with schizophrenia.

Character		N (%)
**Gender**		-
Male		128 (59.3)
Female		88 (40.7)
**Age group (years)**		-
<18		9 (4.2)
18—40		109 (50.5)
41—60		78 (36.1)
>60		20 (9.3)
**Duration of illness**		-
1—3 years		24 (11.1)
4—8 years		32 (14.8)
>8 years		158 (73.1)
**Education level**		-
< High school		-
≥ High school		-
**Number of admissions lifetimes**		-
0		57 (26.4)
1—3		104 (48.1)
4—6		25 (11.6)
>6		30 (13.9)
**Admissions in the past 6 months**		-
Yes		40 (18.5)
No		176 (81.5)
**Taking long-acting injectable antipsychotics**		-
Yes		79 (36.6)
No		137 (63.4)
-		-

**Table 3:** Mean, SD of global and subscale scores.

Subscale	Score Range	Mean	SD
Support for Caring	1—15	7.32	4.67
Caring Choice	1—15	10.76	4.17
Caring Stress	1—15	12.03	3.68
Money Matters	1—15	8.39	3.99
Personal Growth	1—15	8.92	3.82
Sense of Value	1—15	9.86	4.40
Ability to Care	1—15	9.78	3.87
Care Satisfaction	1—15	11.31	3.25
Global	5—120	78.23	21.24

**Table 4:** AC-QoL scores of caregivers with different characteristics.

Variable	Mean (SD)	*t* or *F*	*P*
**Gender/caregiver**	-	-0.035	0.72
Male	77.77 ±23.20	-	-
Female	78.78 ±18.22	-	-
**Age group (years)**	-	-	-
< 45	77.34 ±20.88	-0.60	0.547
> 45	79.12 ±21.65	-	-
**Relationship to the patient**	-	-	-
Parent	77.80 ±20.28	1.14	0.32
Sibling	76.89 ±22.03	-	-
Spouse	77.50 ±22.26	-	-
Child	86.28 ±20.12	-	-
**Education level**	-	-	-
< High school	85.38 ±18.18	2.14	0.03*
≥ High school	76.78 ±21.59	-	-
**Employment status**	-	-	-
Employed	77.78 ±21.40	-0.28	0.77
Unemployed	78.62 ±21.92	-	-
**Time spent in caregiving (per day)**	-	-	-
1—6 hours	81.15 ±19.38	5.30	0.006*
6—12 hours	69.25 ±19.96	-	-
> 12 hours	68.13 ±18.98	-	-
**Duration of illness**	-	-	-
1—3 years	74.20 ±21.06	0.80	0.451
4—8 years	81.46 ±20.46	-	-
>8 years	78.01 ±21.53	-	-
**Number of admissions lifetimes**	-	-	-
0	81.12 ±18.38	1.12	0.341
1—3	78.91 ±21.71	-	-
4—6	73.95 ±17.29	-	-
>6	73.93 ±26.77	-	-
**Admissions in the past 6 months**	-	-	-
Yes	65.60 ±24.6	-3.74	< 0.001*
No	81.21 ±19.27	-	-
**Taking long acting injectable**	-	-	-
Yes	82.16 ±21.21	2.05	0.042*
No	75.96 ±21.00	-	-

## Data Availability

The data and supportive information are available within the article.

## LIST OF ABBREVIATIONS

FCGs: Family caregivers
PwS: Patients with schizophrenia
QoL: Quality of lifes
AC-QoL: Adult Carer Quality of Life scale

## STANDARDS OF REPORTING

STROBE guidelines were followed.
